# Double faecal immunochemical testing in patients with symptoms suspicious of colorectal cancer

**DOI:** 10.1093/bjs/znad016

**Published:** 2023-02-14

**Authors:** A D Gerrard, Y Maeda, J Miller, F Gunn, E Theodoratou, C Noble, L Porteous, S Glancy, P MacLean, R Pattenden, M G Dunlop, F V N Din, Edinburgh Colorectal Group, A Clark, M Collie, D Collins, M Duff, S Goodbrand, J Mander, N Ventham, H Paterson, M Potter, C Reddy, D Speake, F Shaban, G Smith, P Vaughan-Shaw

**Affiliations:** Cancer Research UK Scotland Centre, Institute of Genetics and Cancer, University of Edinburgh, Edinburgh, UK; Department of Colorectal surgery, Western General Hospital, Edinburgh, UK; Cancer Research UK Scotland Centre, Institute of Genetics and Cancer, University of Edinburgh, Edinburgh, UK; Department of Surgery, Queen Elizabeth University Hospital, Glasgow, UK; Department of Colorectal surgery, Western General Hospital, Edinburgh, UK; Department of Colorectal surgery, Western General Hospital, Edinburgh, UK; Cancer Research UK Scotland Centre, Institute of Genetics and Cancer, University of Edinburgh, Edinburgh, UK; Centre for Global Health, Usher Institute, University of Edinburgh, Edinburgh, UK; Department of Gastroenterology, Western General Hospital, Edinburgh, UK; Lead GP for Cancer and Palliative Care, NHS Lothian, Edinburgh, UK; Department of Radiology, Western General Hospital, Edinburgh, UK; Department of Radiology, Western General Hospital, Edinburgh, UK; Department of Biochemistry, Western General Hospital, Edinburgh, UK; Cancer Research UK Scotland Centre, Institute of Genetics and Cancer, University of Edinburgh, Edinburgh, UK; UK Colon Cancer Genetics Group, Medical Research Council Human Genetics Unit, Medical Research Council Institute of Genetics & Cancer, Western General Hospital, The University of Edinburgh, Edinburgh, UK; Cancer Research UK Scotland Centre, Institute of Genetics and Cancer, University of Edinburgh, Edinburgh, UK; Department of Colorectal surgery, Western General Hospital, Edinburgh, UK; Department of Colorectal surgery, Western General Hospital, Edinburgh, UK

## Abstract

**Background:**

Faecal immunochemical test (FIT)-directed pathways based on a single test have been implemented for symptomatic patients. However, with a single test, the sensitivity is 87 per cent at 10 µg haemoglobin (Hb) per g faeces. This aims of this study were to define the diagnostic performance of a single FIT, compared with double FIT in symptomatic populations.

**Methods:**

Two sequential prospective patient cohorts referred with symptoms from primary care were studied. Patients in cohort 1 were sent a single FIT, and those in cohort 2 received two tests in succession before investigation. All patients were investigated, regardless of having a positive or negative test (threshold 10 µg Hb per g).

**Results:**

In cohort 1, 2260 patients completed one FIT and investigation. The sensitivity of single FIT was 84.1 (95 per cent c.i. 73.3 to 91.8) per cent for colorectal cancer and 67.4 (61.0 to 73.4) per cent for significant bowel pathology. In cohort 2, 3426 patients completed at least one FIT, and 2637 completed both FITs and investigation. The sensitivity of double FIT was 96.6 (90.4 to 99.3) per cent for colorectal cancer and 83.0 (77.4 to 87.8) per cent for significant bowel pathology. The second FIT resulted in a 50.0 per cent reduction in cancers missed by the first FIT, and 30.0 per cent for significant bowel pathology. Correlation between faecal Hb level was only modest (*r*_s_ = 0.58), and 16.8 per cent of double tests were discordant, 11.4 per cent in patients with colorectal cancer and 18.3 per cent in those with significant bowel pathology.

**Conclusion:**

FIT in patients with high-risk symptoms twice in succession reduces missed significant colorectal pathology and has an acceptable workload impact.

## Teaser

Single- and double-faecal immunochemical test (FIT) strategies were tested in high-risk symptomatic patients. A double-FIT strategy reduced missed colorectal cancers by 50.0 per cent and all significant bowel pathology by 30.0 per cent, while improving prioritization to investigation. The high proportion of colorectal cancers with discordant FIT results underscores the rationale for double testing. Double FIT could reduce investigation by 68.9 per cent.

## Introduction

Early detection strategies for colorectal cancer, in parallel with preventive measures, are key to improving survival^1^. Colorectal symptoms are a common presentation in primary care; of those referred to secondary care only 3–5 per cent of patients are diagnosed with colorectal cancer^2^. The number of suspected colorectal cancer referrals to secondary care is increasing without a proportionate increase in early cancer diagnoses^3^.

Faecal immunochemical test (FIT) is commonly used to prioritize investigation in symptomatic patients and outperforms symptomatic assessment alone in identifying colorectal cancer^4,5^. Its quantitative nature can optimize resource use by prioritizing those with the highest risk, and tailoring investigation type to the degree of risk and patient characteristics. FIT has been proposed as a gatekeeper investigation in a resource-constrained system, and may even have a role as a rule-out test for colorectal cancer^5^. However, the sensitivity for colorectal cancer at a threshold of 10 µg haemoglobin (Hb) per g in symptomatic patients is unacceptably low (87.2–88.7 per cent)^6–8^. Furthermore, although acceptability studies have suggested that patients favour FIT over colonoscopy at a hypothetical 1 per cent false-negative rate, there was very low acceptability for any test that missed even one additional cancer^9^.

Evidence for the use of more than one FIT to improve test sensitivity is limited and contradictory^10^. A recent retrospective study^11^ in a symptomatic population found a sensitivity of 97.8 per cent when either test value was over 10 µg Hb per g. However, the colorectal cancer prevalence in this study was only 1.1 per cent as the population comprised patients with both low- and high-risk symptoms.

The aim of this study was to define the diagnostic performance of a single FIT compared with testing twice in succession in sequential cohorts of patients referred with symptoms suspicious of colorectal cancer. Diagnostic performance was assessed by measuring overall test performance, the rate of missed colorectal cancers and adenomas, and sensitivity, and implications for workload demand at varying faecal Hb thresholds were considered. A secondary aim was to evaluate whether the results could inform a change in clinical practice.

## Methods

### Study design and participants

The study was designed as two sequential, prospective cohort studies of patients referred consecutively from primary care in South-East Scotland, served by a single National Health Service Trust (Lothian). This captured all urgent or urgent suspected of cancer (USOC) primary care referrals to the Edinburgh Colorectal Surgery Unit. No ethical approval was required as this work formed part of routine care.

Inclusion criteria were USOC or urgent priority referrals with the following high-risk lower gastrointestinal symptoms: repeated rectal bleeding without obvious rectal cause or blood mixed in stool, persistent change in bowel habit, palpable abdominal or rectal mass, weight loss, and/or abdominal pain with or without unexplained iron deficiency anaemia. Exclusion criteria were symptoms not fulfilling the above criteria, known inflammatory bowel disease (IBD), and those undergoing bowel cancer screening or emergency admission investigations.

Patients were allocated to cohorts based on the interval in which their referral was sent. Patients in cohort 1 received a single FIT (single-FIT cohort, January 2019 to February 2020), and those in cohort 2 were sent two FITs on average 13 days apart (double-FIT cohort, March 2020 to July 2021). Patients were encouraged to prepare and return each FIT test on the day of completion. Tests were distributed before colorectal investigation for all patients, and investigation was performed regardless of the FIT being returned or result. Patients in the double-FIT cohort who returned only one FIT before investigation were included in the analysis of the first FIT sample analysed (FIT1) for the double-FIT cohort, whereas those who completed both FITs and colorectal investigation comprise the completed double-test (DT) protocol group. If the second FIT was collected on the same day as the first, it was discounted as a true second test. Endoscopy and CT with colorectal protocols were accepted as colonic/rectal assessment.

Only patients with completed investigations were included in the analysis. However, the electronic patient records of all patients sent a FIT were reviewed, including multidisciplinary team notes and South-East Scotland Cancer Network registry data, at the end of July 2022 (minimum 12-month follow-up, 24 months for the single-FIT cohort) to ensure complete capture of cancer diagnoses in South-East of Scotland. No further cases of colorectal cancer were identified from this review.

### Prospectively recorded data

Demographic data and symptoms at primary care referral were collected prospectively for all patients. High-risk symptoms of abdominal or rectal mass, change in bowel habit (to loose stool or diarrhoea), anaemia, and rectal bleeding were recorded. Hb levels within 3 months were used to evaluate anaemia (less than 135 g/l in men, below 120 g/l in women, in accordance with local laboratory reference range). FIT values and test dates were recorded. All investigation findings were categorized as: normal; colorectal cancer; advanced adenoma (polyp with high-grade dysplasia, at least 10 mm with low-grade dysplasia or sessile serrated lesions with any dysplasia); advanced colorectal neoplasia, which accounted for the presence of either colorectal cancer or advanced adenoma; IBD; and significant bowel pathology, defined as either advanced colorectal neoplasia or IBD.

### Faecal immunochemical test

FIT kits (Hitachi Chemical Diagnostics Systems Co., Ltd, Tokyo, Japan) were mailed to patients with instructions from secondary care and return address envelopes. Samples were analysed at the biochemistry centre using an HM-JACKarc analyser (Hitachi Chemical Diagnostic Systems) which has a reporting limit of quantification (LoQ) of 7 µg Hb per g and a maximum reported result of 400 µg Hb per g. The test positivity threshold was a faecal Hb value of 10 µg Hb per g or greater, consistent with National Institute for Health and Care Excellence (NICE) guidance DG30^12^. The FITTER checklist can be found in *Fig. S1*.

### Data analysis

Quantitative values were compared using χ^2^ test, Fisher’s exact test, or Mann–Whitney *U* test. Correlation was assessed with Spearman’s rank correlation coefficient. Diagnostic accuracy, in terms of positive predictive value, negative predictive value, sensitivity, and specificity, were calculated with 95 per cent confidence intervals. The diagnostic test performance, sensitivity, and workload implications of single- and double-FIT strategies were calculated. Number needed to investigate (NNI; number of patients with a specified FIT result who would need investigating to identify one case of pathology) was determined for bowel pathology at varying thresholds by dividing the number of FIT-positive cases at the given threshold by the cases of bowel pathology within each cohort. Receiver operating characteristic (ROC) curves were generated at different FIT thresholds. Data analysis was performed using R version 4.0.5 (R Foundation for Statistical Computing, Vienna, Austria) with associated packages, and GraphPad Prism (GraphPad Software, San Diego, CA, USA).

## Results

**Fig. 1:**
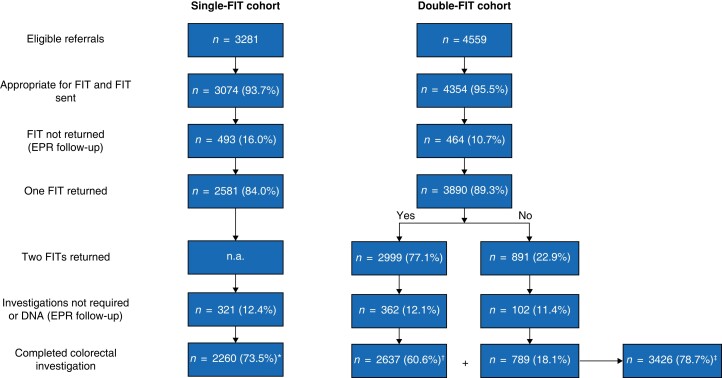
Flow diagram showing single- and double-faecal immunochemical test cohorts from eligible referrals to completed faecal immunochemical test and colorectal investigation *Completed single-faecal immunochemical test (FIT) protocol; †completed double-test protocol; ‡completed at least one FIT. EPR, electronic patient record; n.a., not applicable.

Following consultant triage of eligible general practitioner (GP) referrals received during the study interval, FIT(s) were sent to 93.7 and 95.5 per cent of the single-FIT and double-FIT cohorts respectively (*Fig. 1*). In a minority of patients, based on the GP referral, the triaging consultant considered that FIT was inappropriate, or an emergency admission predated FIT postage. Of the FIT returners, 321 patients (12.4 per cent) in the single-FIT cohort and 464 (11.9 per cent) in the double-FIT cohort did not undergo investigation. This included patients who cancelled investigations, non-attenders, and patients for whom, following consultant review, colorectal investigation was not required. Cancer registry review confirmed that no further colorectal cancers were diagnosed in any patient with minimum 12-month follow-up.

Investigations in the double-FIT cohort (commenced March 2020) were affected by the COVID-19 pandemic. Some 79 per cent of patients in the single-FIT cohort underwent colonoscopy and 21 per cent CT, whereas fewer patients underwent colonoscopy in the double-FIT cohort (53 per cent) than colonic imaging (47 per cent). Although decreased colonoscopy may have reduced adenoma detection in the double-FIT cohort, colorectal cancer detection using CT has been shown to be equivalent to that for colonoscopy^13,14^.

In total, 2260 patients in the single-FIT cohort completed a FIT and colorectal investigation. In the subsequent double-FIT cohort, 3426 patients returned at least one FIT and had colorectal investigation, whereas 2637 completed two FITs (completed DT protocol). The double-FIT cohort included 21 patients from the single-FIT cohort who were re-referred during the double-FIT time frame. Investigation of this group revealed one advanced adenoma (*Table S1*). There was no difference in median age (65 (i.q.r. 56–74) years), sex distribution, FIT positivity, and colorectal cancer or advanced colorectal neoplasia prevalence between cohorts (*Table 1*). There was a lower prevalence of IBD in the double-FIT cohort and this led to a reduced prevalence of overall significant bowel pathology among those who completed the DT protocol, although colorectal cancer and advanced colorectal neoplasia rates were similar. A greater proportion of patients in the single-FIT cohort had anaemia, likely owing to reduced asymptomatic anaemia diagnoses during the COVID phase of double-FIT cohort acquisition, when routine blood tests in primary care ceased.

**Table 1:** Demographics in single- and double-faecal immunochemical test cohorts of patients who had completed the requisite number of tests and undertook colorectal investigation

	Single-FIT cohort completed protocol (*n* = 2260)	Double-FIT cohort	
		Completed at least one FIT (*n* = 3426)	Completed DT protocol (*n* = 2637)
Age (years), median (i.q.r.)	65 (56–74)	65 (56–74)	65 (56–74)
Sex ratio (F : M)	1314 : 946	1909 : 1517	1469 : 1168
Positivity rate test 1 (%)	24.5	24.8	23.8
Additional positivity with test 2 (%)	n.a.	6.3	7.3
Interval from first FIT to investigation (days), median (i.q.r.)	21 (11–43)	23 (13–39)	26 (17–45)*
**Symptom prevalence (%)**			
ȃChange in bowel habit	57.1	60.8	59.8
ȃRectal bleeding	34.7	39.3	37.2
ȃAnaemia	25.1	17.8*	18.2*
ȃAbdominal mass	1.9	3.0*	3.2*
ȃRectal mass	1.6	2.4*	2.4
Colorectal cancer	69 (3.1)	135 (3.9)	88 (3.3)
Advanced adenoma	105 (4.7)	136 (4.0)	97 (3.7)
Advanced colorectal neoplasia	174 (7.7)	271 (7.9)	185 (7.0)
Inflammatory bowel disease	59 (2.6)	55 (1.6)*	33 (1.3)*
Significant bowel pathology	233 (10.3)	326 (9.5)	218 (8.3)*

Values are *n* (%) unless otherwise indicated. Patients may have more than one symptom. FIT, faecal immunochemical test; DT, double test. *P < 0.050 *versus* single-FIT cohort (χ^2^ or Fisher’s exact test for categorical data; Mann–Whitney *U* test for continuous data).

There were 22 colorectal cancers (4.5 per cent) diagnosed among those who did not return a FIT in the single-FIT cohort, and 23 (5.0 per cent) in the double-FIT cohort. Hence, the overall colorectal cancer prevalence in all those appropriate for and sent a FIT, including the non-returners, was 3.0 per cent in the single-FIT cohort and 3.6 per cent in the double-FIT cohort.

### Diagnostic performance of single faecal immunochemical test

At a threshold of 10 µg Hb per g, the sensitivity was 84.1 (95 per cent c.i. 73.3 to 91.8) per cent for detecting colorectal cancer and 64.4 (56.8 to 71.5) per cent for advanced colorectal neoplasia (*Table 2*). If FIT had not been deployed, 33 patients would need investigation to find a colorectal cancer (NNI without FIT: 33). Investigations would be required in 155 patients when the FIT value was below 10 µg Hb per g, compared with 10 patients with the FIT value above the threshold.

**Table 2:** Diagnostic performance in the single-faecal immunochemical test cohort

	*n*	FIT (µg Hb/g)		Sensitivity (%)	Specificity (%)	PPV (%)	NPV (%)	NNI		Pathology missed (%)
		< 10	≥ 10					< 10	≥ 10	
CRC	69	11	58	84.1 (73.3, 91.8)	77.4 (75.6, 79.1)	10.5 (8.0, 13.3)	99.4 (98.8, 99.7)	155	10	15.9
AA	105	51	54	51.4 (41.5, 61.3)	76.8 (75.0, 78.6)	9.7 (7.4, 12.5)	97.0 (96.1, 97.8)	33	10	48.6
ACRN	174	62	112	64.4 (56.8, 71.5)	78.8 (77.0, 80.5)	20.2 (16.9, 23.8)	96.4 (95.4, 97.2)	28	5	35.6
IBD	59	14	45	76.3 (63.4, 86.4)	76.9 (75.1, 78.6)	8.1 (6.0, 10.7)	99.2 (98.6, 99.6)	122	12	23.7
SBP	233	76	157	67.4 (61.0, 73.4)	80.4 (78.6, 82.1)	28.3 (24.6, 32.3)	95.5 (94.5, 96.5)	22	4	32.6

Values in parentheses are 95% confidence intervals. FIT, faecal immunochemical test; Hb, haemoglobin; PPV, positive predictive value; NPV, negative predictive value; NNI, number needed to investigate; CRC, colorectal cancer; AA, advanced adenoma; ACRN, advanced colorectal neoplasia; IBD, inflammatory bowel disease; SBP, significant bowel pathology.

Eleven colorectal cancers were associated with a FIT value below 10 µg Hb per g (*Table S2*) and would have been missed by a single test without investigation. All affected patients were aged over 70 years, eight were anaemic, and six of those with anaemia had a right-sided tumour as did a further two without anaemia. Overall, a single-FIT strategy would miss 15.9 per cent of colorectal cancers and 32.6 per cent of all significant bowel pathology.

### Diagnostic performance of double-faecal immunochemical test strategy

**Fig. 2:**
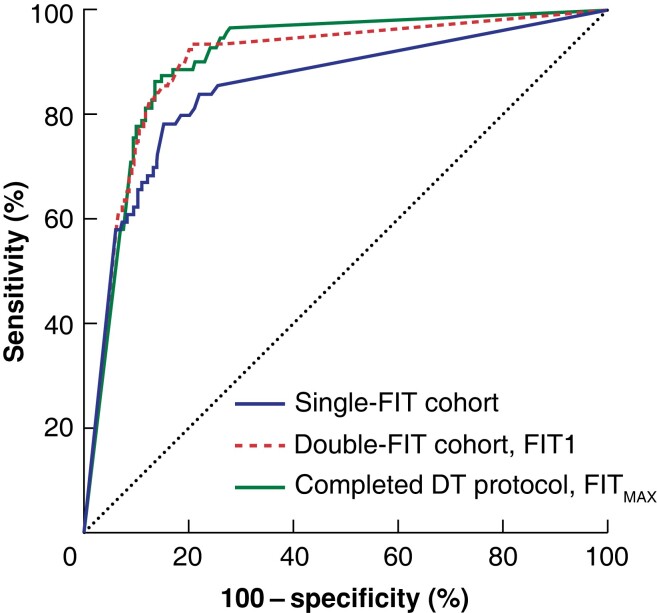
Receiver operating characteristic (ROC) curve analysis for the detection of colorectal cancer between cohorts Area under the curve 0.85 (95% c.i. 0.80 to 0.90) for the single-faecal immunochemical test (FIT) cohort, 0.90 (0.87 to 0.93) for the first FIT analysed (FIT1) of patients who completed at least one test and investigation in the double-FIT cohort, and 0.91 (0.88 to 0.93) for the greatest FIT result (FIT_MAX_) in those who completed the double-test (DT) protocol.

The median interval between completing FITs was 13 (i.q.r. 7–17) days. Some 3426 patients completed the first FIT before colorectal investigation, and 2637 completed both FITs and investigation (completed DT protocol). In the overall double-FIT cohort, the first FIT performed better at identifying colorectal cancer than the previous single-FIT cohort, with a sensitivity of 93.3 (95 per cent c.i. 87.7 to 96.9) per cent (*Fig. 2*). Application of the DT protocol, using the higher of the two FIT results (FIT_MAX_), further increased the sensitivity to 96.6 (90.4 to 99.3) per cent (*Table 3*). Indeed, compared with the single-FIT cohort, completion of the DT protocol resulted in significantly fewer cases of potentially missed colorectal cancer (*P* = 0.009), advanced colorectal neoplasia (*P* < 0.001), and significant bowel pathology (*P* < 0.001). There was a significant improvement in sensitivity for advanced colorectal neoplasia (81.6 (75.3 to 86.9) *versus* 64.4 (56.8 to 71.5) per cent) and significant bowel pathology (83.0 (77.4 to 87.8) *versus* 67.4 (61.0 to 73.4) per cent) in the completed double-FIT compared with the single-FIT cohort.

**Table 3:** Diagnostic performance in double-faecal immunochemical test cohort comparing first analysed test of patients who complete at least one test and investigation with greatest result in those who completed double-test protocol

		*n*	FIT (µg Hb/g)		Sensitivity (%)	Specificity (%)	PPV (%)	NPV (%)	NNI		Pathology missed (%)	Reduction in missed pathology (%)
			< 10	≥ 10					< 10	≥ 10		
CRC	FIT1	135	9	126	93.3 (87.7, 96.9)	78.0 (76.6, 79.4)	14.8 (12.5, 17.4)	99.7 (99.3, 99.8)	286	7	6.7	
	FIT_MAX_	88	3	85	96.6 (90.4, 99.3)	71.2 (69.4, 73.0)	10.4 (8.4, 12.7)	99.8 (99.5, 99.9)	606	10	3.4	49.3
AA	FIT1	136	62	74	54.4 (45.6, 63.0)	76.4 (45.7, 63.0)	8.7 (6.9, 10.8)	97.6 (96.9, 98.2)	42	11	45.6	
	FIT_MAX_	97	31	66	68.0 (57.8, 77.1)	70.4 (68.5, 72.1)	8.1 (6.3, 10.1)	98.3 (97.6, 98.8)	59	12	32.0	29.8
ACRN	FIT1	271	71	200	73.8 (68.1, 78.9)	79.4 (78.0, 80.8)	23.6 (20.7, 26.6)	97.2 (96.5, 97.8)	36	4	26.2	
	FIT_MAX_	185	34	151	81.6 (75.3, 86.9)	72.8 (70.9, 74.5)	18.4 (15.8, 21.3)	98.1 (97.4, 98.7)	53	5	18.4	29.8
IBD	FIT1	55	5	50	90.9 (80.0, 97.0)	76.3 (74.8, 77.7)	5.9 (4.4, 7.7)	99.8 (99.5, 99.9)	515	17	9.1	
	FIT_MAX_	33	3	30	90.9 (75.7, 98.1)	69.7 (67.9, 71.5)	3.7 (2.5, 5.2)	99.8 (99.5, 99.9)	606	27	9.1	0.0
SBP	FIT1	326	76	250	76.7 (71.7, 81.1)	80.7 (79.2, 82.1)	29.4 (26.4, 32.6)	97.1 (96.3, 97.7)	34	3	23.3	
	FIT_MAX_	218	37	181	83.0 (77.4, 87.8)	73.6 (71.8, 75.4)	22.1 (19.3, 25.1)	98.0 (97.2, 98.6)	49	5	17.0	27.0

Values in parentheses are 95% confidence intervals. FIT, faecal immunochemical test; Hb, haemoglobin; PPV, positive predictive value; NPV, negative predictive value; NNI, number needed to investigate; CRC, colorectal cancer; FIT1, first (or only) FIT result; FIT_MAX_, greatest of the two FIT results; AA, advanced adenoma; ACRN, advanced colorectal neoplasia; IBD, inflammatory bowel disease; SBP, significant bowel pathology.

A total of 789 patients completed the first but not the second FIT. The real-world nature of this study meant that investigations were not delayed in order for the second test to be completed. This group were younger, of equal socioeconomic status, and had a colorectal cancer prevalence of 6.0 per cent (*Table S3*).

Internal comparison within the completed DT protocol group, between the first test (FIT1) and maximum result of the two FITs (FIT_MAX_), suggested that adding the second test led to a 50.0 per cent reduction in missed colorectal cancer, 29.2 per cent reduction in advanced colorectal neoplasia, and 30.0 per cent decrease in significant bowel pathology (*Table 4*). Where both FIT values were below 10 µg Hb per g, the prevalence of colorectal cancer was 0.17 per cent, indicating that 606 patients with 2 negative tests would need to be investigated to find 1 additional colorectal cancer. The reassuringly low colorectal cancer prevalence in patients with two negative FITs suggests that urgent investigation could be deferred in lieu of a safety-netting interval of active observation. In the absence of anaemia, the colorectal cancer prevalence with two negative tests was 0.06 per cent with a NNI of 1546.

**Table 4:** Diagnostic performance in those who completed the double-test protocol comparing the first faecal immunochemical test result with the greatest result of two tests

		*n*	FIT (µg Hb/g)		Sensitivity (%)	Specificity (%)	PPV (%)	NPV (%)	NNI		Pathology missed (%)	Reduction in missed pathology (%)
			< 10	≥ 10					< 10	≥ 10		
CRC	FIT1	88	6	82	93.2 (85.7, 97.5)	78.6 (77.0, 80.2)	13.1 (10.5, 16.0)	99.7 (99.4, 99.9)	335	8	6.8	
	FIT_MAX_	88	3	85	96.6 (90.4, 99.3)	71.2 (69.4, 73.0)	10.4 (8.4, 12.7)	99.8 (99.5, 99.9)	606	10	3.4	50.0
AA	FIT1	97	42	55	56.7 (46.3, 66.7)	77.5 (75.8, 79.1)	8.8 (6.7, 11.3)	97.9 (97.2, 98.5)	48	11	43.3	
	FIT_MAX_	97	31	66	68.0 (57.8, 77.1)	70.4 (68.5, 72.1)	8.1 (6.3, 10.1)	98.3 (97.6, 98.8)	59	12	32.0	26.1
ACRN	FIT1	185	48	137	74.1 (67.1, 80.2)	80.0 (78.4, 81.6)	21.9 (18.7, 25.3)	97.6 (96.8, 98.2)	42	5	26.0	
	FIT_MAX_	185	34	151	81.6 (75.3, 86.9)	72.8 (70.9, 74.5)	18.4 (15.8, 21.3)	98.1 (97.4, 98.7)	53	5	18.4	29.2
IBD	FIT1	33	5	28	84.8 (68.1, 94.9)	77.0 (75.4, 78.6)	4.4 (3.0, 6.4)	99.8 (99.4, 99.9)	402	22	15.2	
	FIT_MAX_	33	3	30	90.9 (75.7, 98.1)	69.7 (67.9, 71.5)	3.7 (2.5, 5.2)	99.8 (99.5, 99.9)	606	27	9.1	40.1
SBP	FIT1	218	53	165	75.7 (69.4, 81.2)	80.9 (79.3, 82.5)	26.3 (22.9, 29.9)	97.4 (96.6, 98.0)	38	4	24.3	
	FIT_MAX_	218	37	181	83.0 (77.4, 87.8)	73.6 (71.8, 75.4)	22.1 (19.3, 25.1)	98.0 (97.2, 98.6)	49	5	17.0	30.0

Values in parentheses are 95% confidence intervals. Hb, haemoglobin; FIT, faecal immunochemical test; PPV, positive predictive value; NPV, negative predictive value; NNI, number needed to investigate; CRC, colorectal cancer; FIT1, first FIT result; FIT_MAX_, greatest of the two FIT results; AA, advanced adenoma; ACRN, advanced colorectal neoplasia; IBD, inflammatory bowel disease; SBP, significant bowel pathology.

The use of two FITs improved the likelihood of detecting colorectal cancer in patients and ability to prioritize investigations. The colorectal cancer prevalence was 0.35 per cent when only the first negative FIT of everyone to return at least one test was used, compared with 14.8 per cent if the single FIT was positive. In the double-FIT strategy, the colorectal cancer prevalence was 0.17 per cent where both tests were negative, 2.3 per cent if only one was positive and 19.9 per cent if both had values of 10 µg Hb per g or greater.

### Discordant faecal immunochemical test results

Comparing the FIT results for those with two tests, the correlation was only moderate (*r*_s_ = 0.58, Spearman’s rank correlation) (*Fig. S2*). Overall, 442 patients (16.8 per cent) had discordant FIT results (1 test value 10 µg Hb per g or greater and 1 less, in either order). This occurred both in the presence and absence of significant colorectal pathology (*Table 5*). Of concern, 11.4 per cent of colorectal cancers and 20.5 per cent of advanced colorectal neoplasia had discordant results, which cautions against a single-test strategy whereby this pathology would have been missed.

**Table 5:** Number of discordant results between two faecal immunochemical tests and significant colorectal pathology diagnosed

FIT (µg Hb/g)	Total *n*	Colorectal cancer			Advanced colorectal neoplasia			Inflammatory bowel disease			Significant bowel pathology		
		*n*	Prevalence (%)	NNI	*n*	Prevalence (%)	NNI	*n*	Prevalence (%)	NNI	*n*	Prevalence (%)	NNI
< 10, < 10	1818 (68.9)	3 (3.4)	0.17	606	34 (18.4)	1.90	53	3 (9.1)	0.17	606	37 (17.0)	2.00	49
Discordant	442 (16.8)	10 (11.4)	2.30	44	38 (20.5)	8.60	12	2 (6.1)	0.45	221	40 (18.3)	9.10	11
≥10, ≥ 10	337 (14.3)	75 (85.2)	19.90	5	113 (61.1)	30.00	3	28 (84.8)	7.40	13	141 (64.7)	37.40	3

Values are *n* (%) unless otherwise indicated. FIT, faecal immunochemical test; NNI, number needed to investigate.

**Fig. 3:**
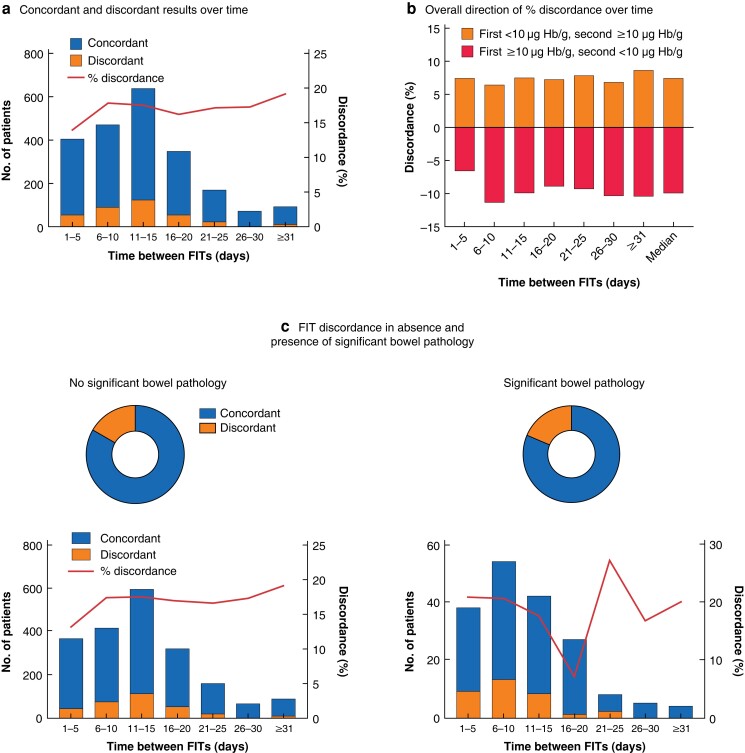
Discordance between first and second faecal immunochemical tests over time and in the absence and presence of significant bowel pathology **a** Total number of patients with concordant and dicordant faecal immunochemical test (FIT) results, and percentage discordance, over time; **b** direction of percentage discordance over time; and **c** FIT discordance according to presence of significant bowel pathology overall (upper panels) and over time (lower panels). There was no significant difference in discordance in the absence *versus* presence of significant bowel pathology (*P* = 0.508, Chi-squared test). Hb, haemoglobin.

To explore whether the interval between FITs influenced discordance levels, discordance was examined at 5-day intervals (*Fig. 3*). The degree of discordance and direction was not obviously affected by the time between samples. There was no trend in separate analyses of those with and without significant bowel pathology.

### Faecal immunochemical test to refine stratification based on symptoms

The double-FIT strategy worked well in all symptoms as a filter to enrich for pathology (*Table S4*). Nonetheless, anaemia remained associated with FIT-negative cancers. The colorectal cancer prevalence in anaemic patients with a single negative FIT was 1.9 per cent, compared with 0.7 per cent in those with two negative FITs. The proportion of anaemic patients with cancers and a negative FIT was similar across the groups: 72.7 per cent in the initial single-FIT cohort, 66.7 per cent in the overall double-FIT cohort, and 66.7 per cent in the completed DT protocol groups where both or the only FIT value was less than 10 µg Hb per g. Of the ten colorectal cancers with discordant FIT results, six were in anaemic patients (*Table S2*). Among patients with diarrhoea, the colorectal cancer prevalence in those with a negative result in the single-FIT cohort was 0.6 per cent, compared with 0.17 per cent in those with two negative FITs. Interestingly, among the 45 patients who had cancers with rectal bleeding as the predominant symptom, none had 2 negative FITs.

### Workload implications of double-faecal immunochemical test strategy

**Fig. 4:**
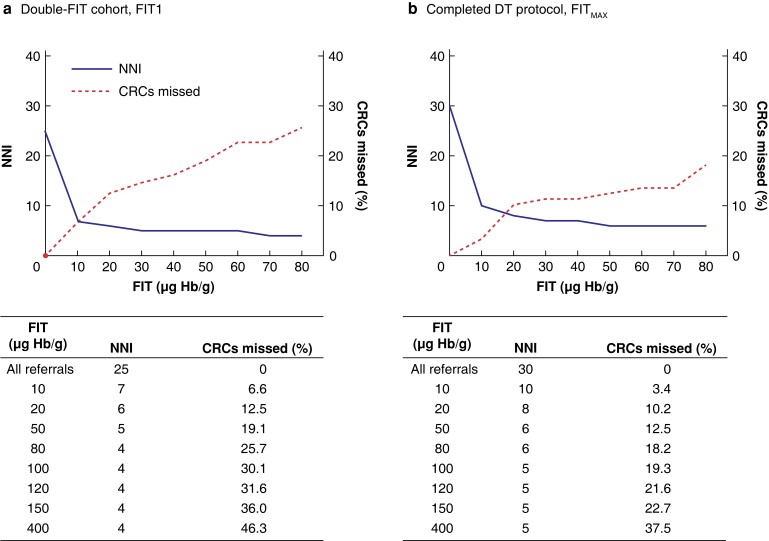
Consequences of increasing the diagnostic threshold on number needed to investigate and percentage of colorectal cancers missed in the double-faecal immunochemical test cohort **a** First sample analysed (FIT1) from all who completed at least one faecal immunochemical test (FIT) and **b** greatest FIT result (FIT_MAX_) for those who completed double-test (DT) protocol. NNI, number needed to investigate; CRC, colorectal cancer; Hb, haemoglobin.

Among those who completed the DT protocol, the positivity was 7.3 per cent higher than when only one test was used. Positivity rate drives workload and is dependent on what threshold for investigation is set. The impact of diagnostic threshold on the NNI and the trade-off in terms of missed pathology is summarized in *Fig. 4*. Raising the threshold from 10 to 20 µg Hb per g resulted in modest reduction in NNI for colorectal cancer but a marked increase in missed colorectal cancers, from 6.6 to 12.5 per cent (FIT1) and 3.4 to 10.2 per cent (FIT_MAX_). Hence, 10 µg Hb per g appears to be the optimal threshold in a double-FIT strategy for balancing workload against detecting colorectal cancer.

**Fig. 5:**
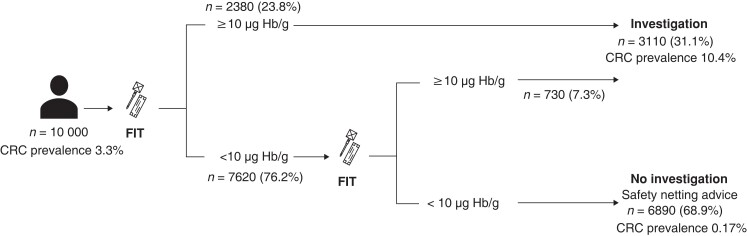
Clinical implementation of double-faecal immunochemical test strategy Values in parentheses are percentage of referrals. Where both faecal immunochemical tests (FITs) show 10 μg haemoglobin (Hb) per g or more, the colorectal cancer (CRC) prevalance is 19.9%.

Modelling of the proposed double-FIT pathway showed that 68.9 per cent of all referrals to secondary care may not require urgent investigation (*Fig. 5*). These patients and their referring practitioner may be reassured by the low colorectal cancer prevalence if both FIT values are below 10 µg Hb per g, particularly in the absence of anaemia.

## Discussion

This study of single- and double-FIT strategies in a high-risk symptomatic population found that double testing reduced missed colorectal cancer and other significant bowel pathology rates, with a modest impact on workload. The diagnostic test performance in the single-FIT cohort was consistent with reported literature^6–8,15^. When two tests were completed in the double-FIT cohort, the sensitivity for colorectal cancer was 96.6 per cent, reducing the missed colorectal cancer rate from the first test by half, and significant bowel pathology by 30.0 per cent. The high proportion of colorectal cancer and significant bowel pathology with discordant results questions the reliability of a single FIT as a rule-out test. Double testing would increase the number of investigations by 7.3 per cent over a single-test approach. Balancing the trade-off between reduced missed pathology and the modestly increased workload suggests that double testing may be worthwhile.

A recent retrospective study^11^ assessed the use of two FITs, comparing outcomes if both, either or neither were positive. Although this was not compared with a single-FIT strategy, the sensitivity for colorectal cancer was 91.5 per cent if both FITs were positive, and 97.8 per cent if either test was positive. The study included low-risk patients, reflected in the lower colorectal cancer prevalence (1.1 per cent). The equally high sensitivity in this wider population supports the use of double testing in low-risk groups. Both this previous work and the present study included referred populations, and are therefore different from unselected patients attending primary care. Increased pathology yield with two positive tests is known to aid colonoscopy resource prioritization^16^. Previous smaller studies have compared one against two FITs in symptomatic patients with mixed results, from no benefit from a second test^17^, to improved sensitivity for colorectal cancer^18^ and advanced colorectal neoplasia^19^. The rates of discordance in these studies varied from 6.1 to 15.3 per cent^11,18^. Previous studies did not systematically report discordance between repeat tests, in the same patient over time, and the relationship to significant bowel pathology. Notable proportions of those with significant bowel pathology had discordant FIT results. The variability occurred consistently over time and irrespective of pathology, presumably owing to intermittent bleeding. Hence, a pragmatic approach of testing a few days apart and ensuring that one stool is not resampled seems appropriate.

The Association of Coloproctology of Great Britain and Ireland and the British Society of Gastroenterology^20^ have recently published guidelines on the use of FIT in symptomatic patients. This study has demonstrated how a second FIT could be positioned to offer a repeat opportunity to reach the diagnostic threshold and mitigate missed colorectal cancer within an acceptable time frame (*Fig. S3*). Those with two negative FITs, following reassessment for ongoing clinical concern, may be directed to alternative pathways.

Strategies to improve single-FIT sensitivity have been explored. Risk stratification models such as COLONOFIT and FAST achieved high sensitivity (98 and 100 per cent respectively), but at the cost of 49.3 and 88 per cent of referrals requiring investigation^21,22^. Lowering the diagnostic threshold to 2 µg Hb per g improves the sensitivity for colorectal cancer detection from 92.2 to 97.7 per cent, but an increased positivity threshold (20.4–39.5 per cent) would adversely affect colonoscopy resource^23^. In this study, FIT enriched sensitivity for colorectal cancer for all common referral symptoms, including rectal bleeding, previously excluded from FIT studies and NICE guidelines in lower-risk symptomatic patients^12,24^, with no colorectal cancers when both FIT values were below 10 µg Hb per g. Conversely, anaemia was associated with a FIT result below 10 µg Hb per g in those with colorectal cancer and colorectal cancer with discordant FIT results. The addition of serum Hb to single FIT raises the sensitivity from 91.8 to 98.3 per cent, but at an increase in NNI to diagnose a colorectal cancer from 18 to 26^25^. This would create a greater workload than the double-FIT strategy without a significant improvement in colorectal cancer detection. Further targeted research into this group is required.

There are limitations to this study. During the double-FIT cohort acquisition, the COVID-19 pandemic resulted in a reduction in endoscopy, with more imaging being performed. The use of CT colonography (CTC) and minimal-preparation CT of the colon (MPCT), used here, has been shown to be comparable to colonoscopy for colorectal cancer diagnosis^15,16^. No subsequent cancers were found in patients undergoing MPCT or CTC with a minimum 12-month follow-up. Although direct comparisons between the single-FIT and double-FIT cohorts are challenging because of inherent differences created by the two time intervals, there are key similarities between the cohorts. Sex and age distributions, FIT1 positivity, and baseline colorectal cancer prevalence were no different. Furthermore, the similar performance of FIT in the single-FIT cohort to published findings suggests that the comparisons of the missed rate of significant colorectal pathology between the cohorts are valid. A second limitation is that not all patients in the double-FIT cohort completed both tests. This was due to planned investigation scheduling at endoscopy, in the real-world context of the study. These patients did not have any greater demographic risk factors for colorectal cancer (*Table S3*), but their increased prevalence of significant bowel pathology justified urgent investigation. The analyser used in this study was an HM-JACKarc with a LoQ of 7 µg Hb per g. This limited the analysis that could be performed at faecal Hb levels below 10 µg Hb per g; comparisons between analysers have reported analytical differences particularly at levels of 4 µg Hb per g and below with OC-Sensor, Eiken Chemical Co., Ltd., Tokyo, Japan (LoQ 2.4 µg Hb per g)^26^.

## Supplementary Material

znad016_Supplementary_Data

## Data Availability

Summarized anonymized data will be made available on request.

## Collaborators

Edinburgh Colorectal Group: A. Clark, M. Collie, D. Collins, M. Duff, S. Goodbrand, J. Mander, N. Ventham, H. Paterson, M. Potter, C. Reddy, D. Speake, F. Shaban, G. Smith, P. Vaughan-Shaw (Western General Hospital, Edinburgh, UK).
