# Supplementary material for: Double faecal immunochemical testing in patients with symptoms suspicious of colorectal cancer
Source: Br J Surg. 2023 Feb 14;110(4):471–80. doi: 10.1093/bjs/znad016 (PMC10364540; doi:10.1093/bjs/znad016)
Supplement: znad016_Supplementary_Data [file znad016_supplementary_data.docx]

**Title:**

Double FIT testing in patients with symptoms suspicious of colorectal cancer

**Authors:**

Gerrard AD^1,2^, Maeda Y^1,3^, Miller J^2^, Gunn F^2^, Theodoratou E^4,5^, Noble C^6^, Porteous L^7^, Glancy S^8^, MacLean P^8^, Pattenden R^9^, Dunlop MG^10,11^, Din FVN^1,2^, Edinburgh Colorectal Group^2^.

^1^ *Institute of Genetics and Cancer, The University of Edinburgh, Edinburgh, Scotland*

^2^ *Department of Colorectal surgery, Western General Hospital, Edinburgh, Scotland*

^3^ *Department of Surgery, Queen Elizabeth University Hospital, Glasgow, Scotland*

^4^ *Centre for Global Health, Usher Institute, The University of Edinburgh, Edinburgh, Scotland*

^5^ *Cancer Research UK centre, Institute of Genetics and Cancer, The University of Edinburgh, Edinburgh, Scotland*

^6^ *Department of Gastroenterology, Western General Hospital, Edinburgh, Scotland*

*^7^ Lead GP for cancer and palliative care, NHS Lothian, Scotland*

^8^ *Department of Radiology, Western General Hospital, Edinburgh, Scotland*

^9^ *Department of Biochemistry, Western General Hospital, Edinburgh, Scotland*

^10^ *Cancer Research UK Edinburgh Centre, Medical Research Council Institute of Genetics & Cancer, Western General Hospital, The University of Edinburgh, Edinburgh, UK*

^11^ *Colon Cancer Genetics Group, Medical Research Council Human Genetics Unit, Medical Research Council Institute of Genetics & Cancer, Western General Hospital, The University of Edinburgh, Edinburgh, UK*

**Corresponding Author**

Farhat Din Reader & Honorary Consultant Colorectal Surgeon

Address: Institute of Genetics and Cancer, University of Edinburgh, Crewe Road, Edinburgh, EH4 2XU

Email: [Farhat.Din@ed.ac.uk](mailto:Farhat.Din@ed.ac.uk)

**Supplementary Materials Index**

**Supplementary Figures and Tables**

Figure S1: FITTER Checklist Pg. 2

Table S1: Re-referred patients in Single and Double-FIT cohorts Pg. 3

Table S2: Colorectal cancers with a FIT less than 10µg Hb/g Pg. 4-5

Figure S2: Double-FIT completed protocol correlation of results Pg. 6

Table S3: Double-FIT cohort one test only vs completed protocol Pg 7

Table S4: FIT performance at stratifying colorectal cancers based on symptoms Pg 8

Figure S3: Proposed integration of second FIT within the ACP/BGS guidelines Pg 9

**Supplementary Figures and Tables**

**Figure S1: FITTER Checklist**

*Specimen collection and handling*

In cohort 1 (January 2019 – February 2020) patients referred to secondary care (3074) were sent one specimen collection device (Hitachi Chemical Diagnostic Systems) and cohort 2 (March 2020 – July 2021) patients (4354) were sent two collection devices on average 13 days apart. The device collects 2mg of faeces with a probe attached to the cap into 2.0ml of buffer. Participants pass the probe into the stool, wrote the date of collection, and returned the collection kits to their local primary care centre who transported the kits to the regional laboratory (Dundee, Scotland). In total, 6471 patients completed at least one FIT.

*Analysis*

Specimens were analysed using the HM-JACKarc analyser. Samples were stored at 4°C until the time of analysis and allowed to warm to room temperature. The analytical working range in 7-400µg Hb/g.

*Quality management*

All analysis is carried out at the regional laboratory. There are daily internal quality control measures with repeated sampling and between batch impression aiming for ±2SD. External quality assessment is performed three times per month with sample material prepared by EQU laboratories (Birmingham, England).

*Data Handling*

The f-Hb concentrations are recorded electronically and linked back to the patient record to be available to the clinical team.

**Table S1: Re-referred patients in Single and Double-FIT cohorts**

| Single-FIT cohort | | | Double-FIT cohort | | | |
| --- | --- | --- | --- | --- | --- | --- |
| **Symptoms** | **FIT (µg Hb/g)** | **Investigation Finding** | **Symptoms** | **FIT (µg Hb/g)** | **FIT (µg Hb/g)** | **Investigation Finding** |
| Anaemia, CIBH | 23 | Polyp | CIBH | 0 | 0 | Normal |
| Anaemia, PR Bleeding | 400 | Haemorrhoids | CIBH | 400 | 0 | Diverticulosis |
| PR Bleeding | 0 | Haemorrhoids | PR Bleeding | 0 | 0 | Haemorrhoids |
| CIBH | 0 | Polyp | CIBH | 0 | 0 | Normal |
| Anaemia, PR Bleeding | 0 | Normal | PR Bleeding | 0 | 0 | Diverticulosis |
| CIBH | 0 | Polyp | Possible Rectal Mass | 0 | 0 | Normal |
| Diarrhoea | 0 | Polyp | PR Bleeding | 0 | 0 | Normal |
| CIBH | 121 | Normal | Constipation | 21 | 32 | Normal |
| Anaemia | 0 | Normal | CIBH | 0 | 0 | Advanced Adenoma |
| Anaemia | 15 | Normal | CIBH | 400 | 0 | Nicorandil induced ulceration |
| CIBH | 35 | Diverticulosis | CIBH | 0 | 0 | Diverticulosis |
| PR Bleeding | 0 | Polyp | CIBH | 0 | 0 | Diverticulosis |
| PR Bleeding | 8 | Normal | PR Bleeding | 400 | 127 | Diverticulosis |
| Anaemia | 14 | Polyp | CIBH | 11 | 63 | Haemorrhoids |
| CIBH | 0 | Normal | CIBH | 0 | 15 | Normal |
| CIBH | 311 | Polyp | PR Bleeding | 400 | 21 | Normal |
| PR Bleeding | 0 | Haemorrhoids | Abdominal Pain | 0 | 0 | Normal |
| CIBH | 0 | Advanced Adenoma | CIBH | 0 | 0 | Normal |
| Abdominal Pain | 0 | Normal | Abdominal Pain | 107 | 19 | Normal |
| CIBH | 0 | Normal | CIBH | 0 | 0 | Normal |
| CIBH | 0 | Diverticulosis | Abdominal Pain | 0 | 0 | Diverticulosis, Polyp |

CIBH: Change in bowel habit to looser/frequent stool

**Table S2: Colorectal cancers with a FIT less than 10µg Hb/g**

1. Single-FIT cohort CRC f-Hb less than 10µg Hb/g

| Age | Sex | Primary Symptoms | FIT (µg Hb/g) | Hb (g/L) | Primary CRC Site | Tumour Morphology | T Stage |
| --- | --- | --- | --- | --- | --- | --- | --- |
| 71 | F | Anaemia | 0 | 85 | Hepatic flexure | Stricturing | 4 |
| 71 | F | Anaemia | 0 | 111 | Transverse | Polypoidal | 1 |
| 76 | M | Loose stool | 0 | 137 | Transverse | Ulcerative | 3 |
| 76 | M | PR Bleed | 0 | 137 | Rectum | Ulcerative | 3 |
| 77 | F | Anaemia | 0 | 108 | Transverse | Stricturing | 3 |
| 78 | F | Loose stool | 0 | 105 | Sigmoid | Stricturing | 4 |
| 78 | F | Diarrhoea | 0 | 139 | Caecum | n/a | 4 |
| 84 | M | Diarrhoea, Thrombocytopaenia | 0 | 132 | Rectum | Polypoidal | 1 |
| 85 | F | Anaemia | 0 | 100 | Caecum | n/a | 4 |
| 86 | F | Anaemia | 0 | 124 | Ascending colon | Polypoidal | 2 |
| 86 | M | Diarrhoea | 7 | 124 | Transverse | Polypoidal | 3 |

Shaded = Patients with anaemia (Male Hb <135 g/L, Female <120 g/L)

1. Complete DT protocol CRC f-Hb less than 10µg Hb/g

| Age | Sex | Primary Symptoms | FIT1 (µg Hb/g) | FIT2 (µg Hb/g) | Hb (g/L) | Primary CRC Site | Tumour Morphology | T Stage |
| --- | --- | --- | --- | --- | --- | --- | --- | --- |
| 73 | F | Loose stool | 0 | 0 | 135 | Rectum | Polypoidal | 1 |
| 77 | M | Diarrhoea, Anaemia | 0 | 0 | 123 | Splenic flexure | Stricturing | 3 |
| 80 | F | Loose stool | 0 | 0 | 106 | Sigmoid | Stricturing | x |

Shaded = Patients with anaemia (Male Hb <135 g/L, Female <120 g/L)

1. Discordant FIT CRC

| Age | Sex | Primary Symptoms | FIT (µg Hb/g) | FIT (µg Hb/g) | Hb (g/L) | Primary CRC Site | Tumour Morphology | T Stage |
| --- | --- | --- | --- | --- | --- | --- | --- | --- |
| 60 | F | Loose stool, PR Bleed | 14 | 0 | 139 | Rectum | Polypoidal | 2 |
| 65 | M | Loose stool, PR Bleed | 400 | 0 | 143 | Sigmoid | Polypoidal | 3 |
| 69 | M | Abdo pain, Anaemia | 58 | 0 | 100 | Caecum | n/a | x |
| 76 | M | Diarrhoea | 0 | 11 | 133 | Transverse | Polypoidal | 1 |
| 76 | F | Diarrhoea | 19 | 0 | 134 | Sigmoid | Polypoidal | 4 |
| 80 | F | PR Bleed | 400 | 0 | 118 | Rectum | Stricturing | 2 |
| 80 | M | Loose stool | 0 | 205 | 127 | Rectosigmoid | Stricturing | 4 |
| 82 | F | Diarrhoea | 40 | 8 | 96 | Ascending colon | n/a | x |
| 83 | F | Anaemia | 12 | 0 | 96 | Caecum | Polypoidal | 4 |
| 84 | F | Diarrhoea, PR Bleed | 0 | 24 | 128 | Rectum | Polypoidal | 3 |

Shaded = Patients with anaemia (Male Hb <135 g/L, Female <120 g/L)

1. Double-FIT cohort with only one test returned and FIT<10µg Hb/g

| Age | Sex | Primary Symptoms | FIT (µg Hb/g) | FIT (µg Hb/g) | Hb (g/L) | Primary CRC Site | Tumour Morphology | T Stage |
| --- | --- | --- | --- | --- | --- | --- | --- | --- |
| 74 | F | Loose stool | 0 | - | 133 | Transverse colon | Polypoidal | 3 |
| 78 | M | Anaemia | 0 | - | 89 | Caecum | Polypoidal | 4 |
| 84 | F | Loose stool, Anaemia | 0 | - | 115 | Caecum | Polypoidal | 2 |

Shaded = Patients with anaemia (Male Hb <135 g/L, Female <120 g/L)

**Figure S2: Double-FIT completed protocol correlation of results**

1. Time between completed tests

Median: 13 days

IQR:7-17 days

1. Correlation of FIT value between FIT1 and FIT2

Spearman Rank Correlation 0.58 (0.55-0.60)

1. Correlation of FIT positivity between FIT1 and FIT2

|  |  | FIT 2 | |
| --- | --- | --- | --- |
|  |  | <10 | ≧10 |
| FIT1 | <10 | 1818 (68.9%) | 192 (7.3%) |
|  | ≧10 | 250 (9.5%) | 377 (14.3%) |

**Table S3: Double-FIT cohort one test only vs completed protocol**

|  | **Only FIT 1** | **Completed DT protocol** | ***p-value*** |
| --- | --- | --- | --- |
| Number with requisite FITs and colorectal investigations | 789 | 2637 |  |
|  |  |  |  |
| Median age (IQR) | 62 (54-72) | 65 (56-74) | *<0.001* |
| Sex, F (%) | 439 (55.6) | 1469 (55.7) | *0.973* |
| Scottish Index of Multiple Deprivation (median, IQR) | 7 (4-9) | 7 (4-9) | *0.132* |
|  |  |  |  |
| CRC (%) | 47 (6.0) | 88 (3.3) | *0.001* |
| AA (%) | 39 (4.9) | 97 (3.7) | *0.111* |
| ACRN (%) | 86 (10.9) | 185 (7.0) | *<0.001* |
| IBD (%) | 22 (2.8) | 33 (1.3) | *0.003* |
| SBP (%) | 108 (13.7) | 218 (8.3) | *<0.001* |

CRC; Colorectal cancer, AA; Advanced adenoma, ACRN; Advanced colorectal neoplasia, IBD; Inflammatory bowel disease, SBP; Significant bowel pathology. Only FIT1; Double-FIT cohort only to complete one FIT prior to colorectal investigation, Completed DT protocol; Double-FIT cohort completed two FITs and colorectal investigation

**Table S4: FIT performance at stratifying CRC based on symptoms**

1. Single-FIT cohort

|  | CIBH | | PR bleeding | | Anaemia | | Abdominal Mass | | Rectal Mass | |
| --- | --- | --- | --- | --- | --- | --- | --- | --- | --- | --- |
| Total Number of patients (%) | 57.1% | | 34.7% | | 25.1% | | 1.9% | | 1.6% | |
| CRC Prevalence (%) | 3.0% | | 3.8% | | 6.7% | | 4.7% | | 11.4% | |
|  |  |  |  |  |  |  |  |  |  |  |
| **FIT (µg Hb/g)** | **<10** | **≧10** | **<10** | **≧10** | **<10** | **≧10** | **<10** | **≧10** | **<10** | **≧10** |
| Number of patients (%) | 77.0% | 23.0% | 67.3% | 32.7% | 65.3% | 34.7% | 72.1% | 27.9% | 74.3% | 25.7% |
| CRC Prevalence (%) | 0.6% | 11.1% | 0.2% | 11.3% | 1.9% | 15.7% | 0.0% | 16.7% | 0.0% | 44.4% |

1. Completed DT protocol

|  | CIBH | | PR bleeding | | Anaemia | | Abdominal Mass | | Rectal Mass | |
| --- | --- | --- | --- | --- | --- | --- | --- | --- | --- | --- |
| Total Number of patients (%) | 59.8% | | 37.2% | | 18.2% | | 3.2% | | 2.4% | |
| CRC Prevalence (%) | 3.4% | | 4.7% | | 6.1% | | 1.2% | | 16.1% | |
|  |  |  |  |  |  |  |  |  |  |  |
| **FIT_MAX_ (µg Hb/g)** | **<10** | **≧10** | **<10** | **≧10** | **<10** | **≧10** | **<10** | **≧10** | **<10** | **≧10** |
| Number of patients (%) | 71.7% | 28.3% | 56.5% | 43.5% | 56.8% | 43.2% | 79.8% | 20.2% | 56.5% | 43.5% |
| CRC Prevalence (%) | 0.3% | 11.4% | 0.0% | 10.8% | 0.7% | 13.0% | 0.0% | 5.9% | 0.0% | 37.0% |

CRC; Colorectal cancer, CIBH; Change in bowel habit to looser stool and/or diarrhoea, patients may have more than one symptom, FIT_MAX_ greatest of the two FIT

Figure S3: Proposed integration of second FIT within the ACP/BGS guidelines
